# Supplementary material for: C2H2 Zinc-Finger Transcription Factors Coordinate Hormone–Stress Crosstalk to Shape Expression Bias of the Flavonoid Pathway in Safflower (Carthamus tinctorius L.)
Source: Curr Issues Mol Biol. 2025 Dec 8;47(12):1023. doi: 10.3390/cimb47121023 (PMC12731760; doi:10.3390/cimb47121023)
Supplement: Supplementary file 1 [file cimb-47-01023-s001.zip › Supplementary Figure S2 Dissociation curve analysis for qPCR primer specificity validation.pdf]

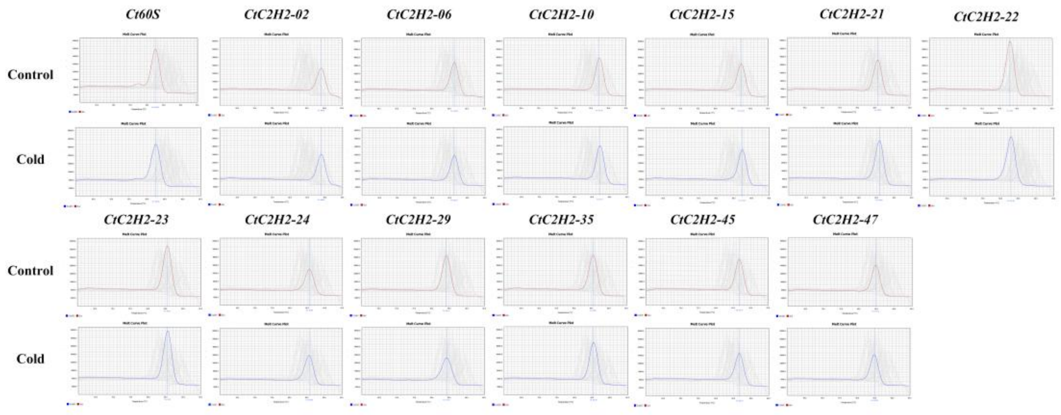

Figure S2. Dissociation curve analysis for qPCR primer specificity validation. The presence of a single, sharp peak for each primer pair indicates specific amplification of a single PCR product. The analysis was performed with three technical replicates for each primer set; one representative replicate is shown for clarity.
