# Supplementary figures and images for: C2H2 Zinc-Finger Transcription Factors Coordinate Hormone–Stress Crosstalk to Shape Expression Bias of the Flavonoid Pathway in Safflower (Carthamus tinctorius L.)
Source: Curr Issues Mol Biol. 2025 Dec 8;47(12):1023. doi: 10.3390/cimb47121023 (PMC12731760; doi:10.3390/cimb47121023)

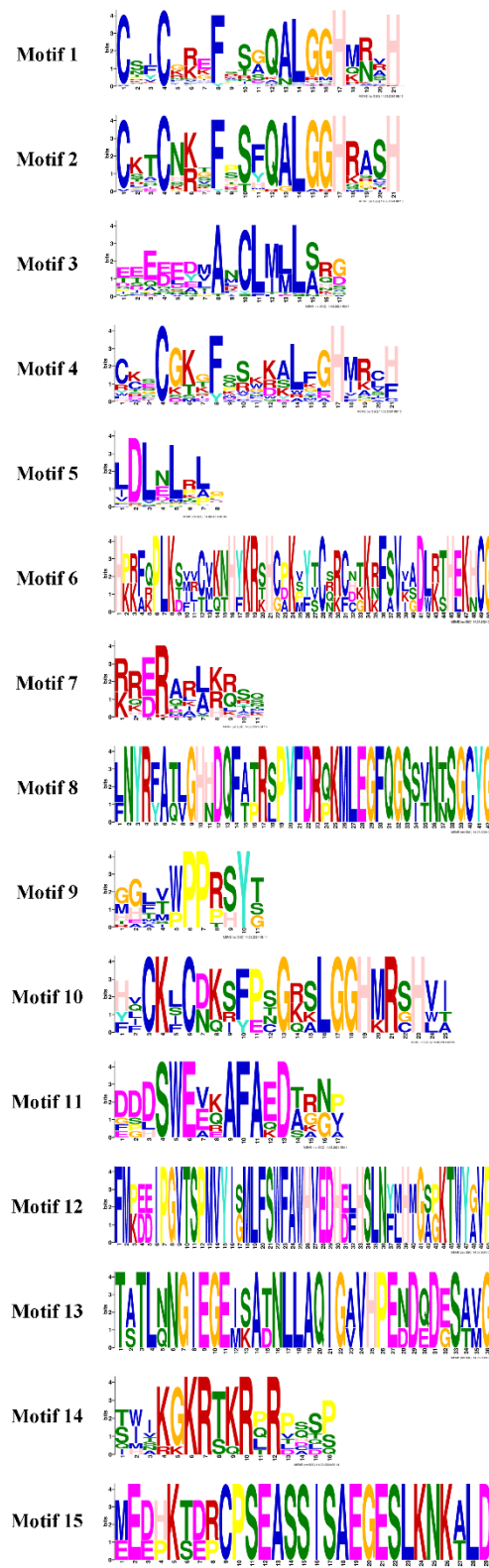

**Figure S1.** Conserved motif 1-15. 62 conserved motifs of the *CtC2H2* gene, comprising 15 motifs (Motif 1 to Motif 15)

Supplement: Supplementary file 1 [file cimb-47-01023-s001.zip › Supplementary Figure S1 Conserved motif 1-15.pdf]
